# Essential Domains of Oxysterol-Binding Protein Required for Poliovirus Replication

**DOI:** 10.3390/v14122672

**Published:** 2022-11-29

**Authors:** Minetaro Arita

**Affiliations:** Department of Virology II, National Institute of Infectious Diseases, 4-7-1 Gakuen, Musashimurayama-shi, Tokyo 208-0011, Japan; minetaro@niid.go.jp; Tel.: +81-42-561-0771; Fax: +81-42-561-4729

**Keywords:** virus, picornavirus, enterovirus, OSBP, PI4KB

## Abstract

Oxysterol-binding protein (OSBP) is a host factor required for enterovirus (EV) replication. OSBP locates at membrane contact site and acts as a lipid exchanger of cholesterol and phosphatidylinositol 4-phosphate (PI4P) between cellular organelles; however, the essential domains required for the viral replication remain unknown. In this study, we define essential domains of OSBP for poliovirus (PV) replication by a functional dominance assay with a series of deletion variants of OSBP. We show that the pleckstrin homology domain (PHD) and the ligand-binding domain, but not the N-terminal intrinsically disordered domain, coiled-coil region, or the FFAT motif, are essential for PV replication. The PHD serves as the primary determinant of OSBP targeting to the replication organelle in the infected cells. These results suggest that not all the domains that support important biological functions of OSBP are essential for the viral replication.

## 1. Introduction

Enterovirus (EV) is a small non-enveloped virus with a positive-sense single-stranded RNA genome of about 7500 nt belonging to the family *Picornaviridae*, including poliovirus (PV, species *Enterovirus C*) [1].

Positive-sense single-stranded RNA viruses, including EV, remodel cellular organelles into viral replication organelles (ROs) by modulating the lipid homeostasis in the infected cells (reviewed in [2]). Host phosphatidylinositol-4 kinases PI4KA and PI4KB have been identified as host factors for the replication of hepatitis C virus (HCV, the family *Flaviviridae*) [3,4] and EV [5], respectively, which are required for the formation of the viral replication complex. Subsequently, oxysterol-binding protein (OSBP) family I (OSBP and OSBP2/ORP4) was identified as the target of a group of antiviral candidates [6,7], which forms a functional axis with PI4KB/PI4KA in the viral replication [8,9].

OSBP (GenBank: NM_002556, 807 amino acid residues), which was originally identified as a high-affinity receptor for oxysterol, [10], acts as an exchanger of cholesterol/phosphatidylinositol 4-monophosphate (PI4P) [11,12], resulting in transport of cholesterol from endoplasmic reticulum (ER) to the *trans* Golgi [12] and to lysosomes for rapid lysosomal repair [13,14] and from recycling endosomes to the *trans* Golgi [15]. OSBP could transport PI4P to the plasma membrane via dimerization with OSBP2 in T cells [16].

In PV-infected cells, the functional axis of PI4KB/ OSBP, thus production of PI4P by PI4KB and coupled accumulation of unesterified cholesterol (UC) by OSBP in the RO, is essential for cleavage of the viral 3AB protein by the viral protease for development of RO and synthesis of the viral plus-strand RNA [17,18,19,20]. The viral 2B protein is essential to enhance the viral replication and the growth by complementing the axis after the cleavage of the 3AB protein; however, the mechanism remains largely unknown [21].

OSBP consists of at least five functional domains: an *N*-terminal intrinsically disordered region (IDR, residues 1–90) [22], a pleckstrin homology domain (PHD, residues 91–179), a coiled-coil region (CCR, residues 180–349) including the dimerization domain (residues 261–288) [12,23], two phenylalanines in an acidic tract (FFAT) motif (residues 350–360) [24], and a ligand-binding domain (OSBP-related domain, ORD, residues 410–807) [12,25]. Exact functions and domains of OSBP required for viral replication remain unknown.

Recently, our group solved a crystal structure of the ORD of human OSBP [26]. Based on the crystal structure, we developed a novel functional dominance assay of OSBP using the M446W variant that could confer tolerance to an OSBP inhibitor T-00127-HEV2 [6,26], which inhibits the sterol transfer activity of endogenous WT OSBP [19]. Since OSBP serves as an essential gene in human cell lines [27], *trans* complementation assay with the knockout cells is not applicable for the characterization of OSBP. The functional dominance assay allows evaluation of in vivo function of OSBP by using ectopically expressed OSBP variants without using the knockout cells. In the present study, we characterize deletion variants of OSBP and define the essential domains of OSBP required for PV replication.

## 2. Materials and Methods

**Cells.** HEK293 cells (human embryonic kidney cells) were cultured as monolayers in Dulbecco’s modified Eagle medium (DMEM, FUJIFILM Wako Pure Chemical Corporation, Osaka 044-29765, Japan) supplemented with 10% fetal calf serum (FCS). GP2-293 cells (Takara Bio USA, Inc., San Jose, CA, USA) were used for retrovirus production.

**Viruses.** PV1_pv_ was produced with a firefly-luciferase-encoding or a mCherry-encoding type 1 PV (Mahoney) (GenBank: V01149) replicon and capsid proteins of PV1(Mahoney) (PV1[Fluc]_pv_ or PV1[mCherry]_pv_, respectively) [28]. 

**Chemicals.** An OSBP inhibitor, T-00127-HEV2 ((3β,17β)-16,16-dimethyl-3-[(tetrahydro-2H-pyran-2-yl)oxy]-Androst-5-en-17-ol), was kindly provided by Hirotatsu Kojima (Drug Discovery Initiative, The University of Tokyo, Japan). The purity of compounds was determined by LC-MS based on the signal of Evaporative Light Scattering Detection. The purity of T-00127-HEV2 was >99%.

**General methods for molecular cloning.***Escherichia coli* strain XL10gold (Agilent Technologies, Inc., Santa Clara, CA, USA) was used for the preparation of plasmids. Ligation of DNA fragments was performed using an In-Fusion HD Cloning Kit (Takara Bio USA, Inc.). PCR was performed using KOD Plus DNA polymerase (TOYOBO CO., LTD., Osaka, JPN). DNA sequencing was performed using a BigDye Terminator v3.1 cycle sequencing ready reaction kit (Thermo Fisher Scientific Inc., Waltham, MA, USA) and then analyzed with a 3500xL genetic analyzer (Thermo Fisher Scientific Inc.).

**Plasmids**

**mCherry-encoding PV(Mahoney) replicon.** A plasmid-encoding, mCherry-encoding PV replicon was constructed by ligation of a DNA fragment of a plasmid-encoding cDNA of a PV replicon (pPV-Fluc mc) [29], followed by addition of a hammerhead ribozyme at the 5′ end of the replicon [30]. The DNA fragment of the plasmid was obtained by PCR by deleting the firefly-luciferase-coding region by using pPV-Fluc mc as the template and prime set 1. The cDNA fragment of mCherry gene was obtained by PCR by using a cDNA of mCherry gene (a kind gift from Ryuichi Sugiyama) as the template and primer set 2. A hammerhead ribozyme was added to the 5′ end of the replicon by PCR with primer set 3 followed by self-ligation. The resulting construct was designated pPV-mCherry mc.

Primer set 1:

5′-GCCAAGAAGGGCGGAAAGTCCAAATTGGAG-3′

5′-CGGGCCTTTCTTTATGTTTTTGGCGTCTTC-3′

Primer set 2:

5′-ATAAAGAAAGGCCCGGTGAGCAAGGGCGAGGAGGATAAC-3′

5′-TCCGCCCTTCTTGGCGTCCATGCCGCCGGTGGAGTGGCG-3′

Primer set 3:

5′-CGGTATCCCGGGTTCTTAAAACAGCTCTGGGGTTGTACCC-3′

5′-GAACCCGGGATACCGGGTTTTCGGCCTTTCGGCCTCATCAGTTAAAACACCCTATAGTGAGTCGTATTAATTTCGATAAGCC-3′

**OSBP-EGFP expression vectors.** A retroviral expression vector for human OSBP-EGFP were constructed previously with pLEGFP-N1 (Takara Bio USA, Inc., CA, USA) (pLEGFP-N1-hOSBP[WT] and pLEGFP-N1-hOSBP[M446W]) [6,26]. The expression vectors for OSBP variants were constructed by PCR using pLEGFP-N1-hOSBP(M446W) as the template, with primer sets (below) followed by self-ligation [26]. For the production of the retrovirus, GP2-293 cells were co-transfected with the expression vectors and pVSV-G (Takara Bio USA, Inc.), and then the cell culture supernatant of the transfected cells was collected at 48 and 72 h p.t. HEK293 cells were infected with the retrovirus vectors and then used for the assays.

Primer set for Δ2–90:

5′-GAGCCAGCCCTCTCGCATGGTAAGCTTGAGCTCGAGATC-3′

5′-CGAGAGGGCTGGCTCTTCAAATGG-3′

Primer set for Δ2–405:

5′-TGGTATTCTGGTTCTCATGGTAAGCTTGAGCTCGAGATC-3′

5′-AGAACCAGAATACCATACAAGCCAAAC-3′

Primer set for Δ91–179:

5′-AGCCGAGCCCGAACCCCCAGCGCCCGAGCC-3′

5′-GGTTCGGGCTCGGCTAAGGCCAAAGCTGTGAAGATGCTGG-3′

Primer set for Δ180–349:

5′-GGCCAGTTCCAGGGCCGTCACCCAGCGC-3′

5′-GCCCTGGAACTGGCCATGAGCGATGAAGATGATGAGAATG-3′

Primer set for Δ180–360:

5′-GGCCAGTTCCAGGGCCGTCACCCAGCGC-3′

5′-GCCCTGGAACTGGCCGATGCACCTGAGATCATCACCATGC-3′

Primer set for Δ350–360:

5′-GTCCCCTTTGCCAGAGCAGCACTGATC-3′

5′-TCTGGCAAAGGGGACGATGCACCTGAGATCATCACCATGCC-3′

Primer set for Δ754–807:

5′-GGCGACCGGTGGATCTTCCGCTTCTCTCTTCTTTCTGGAAAG-3′

5′-GATCCACCGGTCGCCACCATGGTGAGCAAG-3′

**Microscopy.** OSBP-EGFP-expressing HEK293 cells (8 × 10^3^ cells/well) were plated into a 384-well PhenoPlate-384 (PerkinElmer, Waltham, MA, USA, 6057302). The cells were infected with PV1(mCherry)_pv_ at a multiplicity of infection (MOI) of 50 for 7 h at 37 °C and then fixed with 3% paraformaldehyde for 10 min at room temperature. Images were collected at 60× magnification using FV3000 confocal microscopy (Olympus Corporation, Tokyo, Japan). Maximum projection images of cells were reconstructed from the confocal images obtained at intervals of about 0.36 μm along the optical *z*-axis (about 30 confocal sections for each maximum projection image). 

**Flow cytometry.** OSBP-EGFP-expressing HEK293 cells were suspended in DMEM supplemented with 10% FCS. About 5.0 × 10^4^ cells were measured per sample with a BD FACSLyric Flow Cytometer (BD, Franklin Lakes, NJ, USA). Data were analyzed using FlowJo software (BD). Geometric means of EGFP signals and the percentage of OSBP-EGFP-expressing cells were quantified.

**Functional dominance assay of OSBP**. HEK293 cells expressing the OSBP variants (9.6 × 10^3^ cells per well in 20 μL medium) in 384-well plates (Greiner Bio-One, 781080) were inoculated with 10 μL of PV1(Fluc)_pv_ (4.8 × 10^3^ infectious units [IU]) and 10 μL of 0 or 80 μM T-00127-HEV2 solution (final concentrations of 0 or 20 μM). Cells were incubated at 37 °C for 7 h. Luciferase activity in the infected cells was measured with the Steady-Glo luciferase assay system (Promega Corporation, Fitchburg, WI, USA) using a 2030 ARVO X luminometer (PerkinElmer). Luciferase activity of the infected cells was measured at 7 h post-infection (p.i.) with a Steady-Glo Luciferase Assay System (Promega Corporation). Luciferase activity in PV1(Fluc)_pv_-infected cells in the absence of the compound was taken as 100%.

**Western blot.** HEK293 cells expressing the OSBP variants (8.0 × 10^5^ cells) were collected in 100 μL cell lysis buffer (21 mM HEPES buffer [pH 7.4], 0.7 mM disodium hydrogenphosphate, 137 mM NaCl, 4.8 mM KCl, 0.5% Nonidet P-40, and 5 mM EDTA, supplemented with complete mini protease inhibitor cocktail tablet [Roche, 04 693 159 001]) and then were subjected to e-PAGEL 5–20% gradient polyacrylamide gel electrophoresis (ATTO CORPORATION, Tokyo, Japan) in a Laemmli buffer system. Proteins in the gel were transferred to a polyvinylidene difluoride filter (MilliporeSigma, Burlington, MA, USA, Immobilon) and blocked in iBind solution (Thermo Fisher Scientific Inc.). Filters were incubated with anti-GFP antibody (MEDICAL & BIOLOGICAL LABORATORIES CO., LTD., Tokyo, Japan, 598, rabbit polyclonal antibody, 1:2,000 dilution) then with secondary antibodies (Thermo Fisher Scientific Inc., goat anti-rabbit IgG antibodies conjugated with horseradish peroxidase, 1:200 dilution) in an iBind Western System (Thermo Fisher Scientific Inc.). Signals were detected with SuperSignal West Femto Maximum Sensitivity Substrate (Thermo Fisher Scientific Inc.) then analyzed with Amersham ImageQuant 800 (Cytiva, Tokyo, Japan).

**Statistical analysis.** The results of the experiments are shown as means with standard deviations. Values of *P* < 0.05 by one-tailed *t* test were considered to indicate a significant difference and were indicated by asterisks (* *P* < 0.05, ** *P* < 0.01, *** *P* < 0.001).

## 3. Results

**Figure 1:**
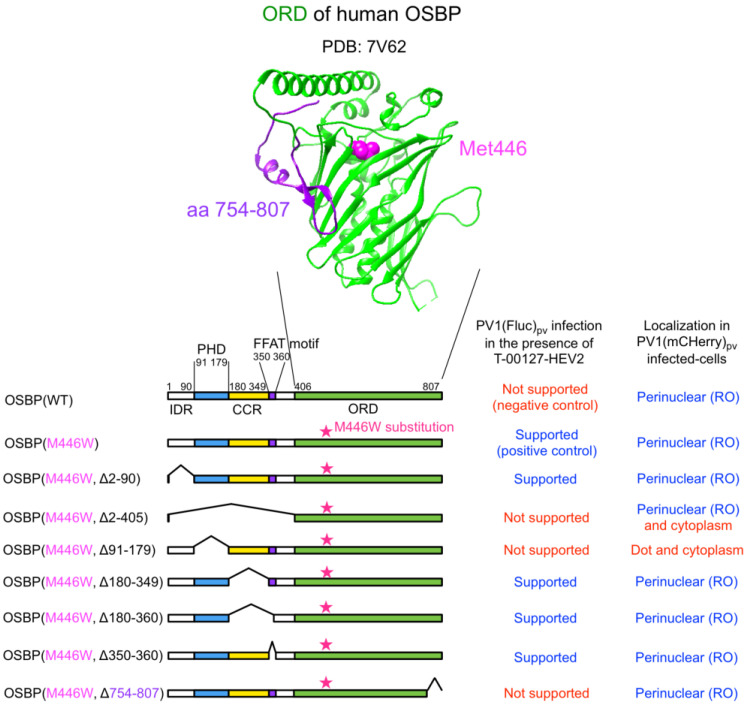
OSBP variants analyzed in this study. (**Upper**) A crystal structure of ligand-binding domain of human OSBP (PDB: 7V62). The M446 residue and a C-terminal region (aa 754–807) of the OSBP is highlighted in magenta and purple, respectively. (**Lower**) Domain organizations of OSBP variants analyzed in this study. The corresponding amino acid numbers of the domains are shown. IDR: intrinsically disordered region; PHD: pleckstrin homology domain; CCR: coiled-coil region; FFAT: two phenylalanines in an acidic tract.

We generated a series of HEK293 cell lines overexpressing the M446W variants of C-terminally EGFP-fused OSBP (OSBP-EGFP) with deletions of each functional domain (Figure 1). For the ORD-deletion variant, partial deletion was introduced in a conserved C-terminal region (aa 754–807) to give a functionally null variant with the M446W substitution (a negative control of the M446W variant series). 

**Figure 2:**
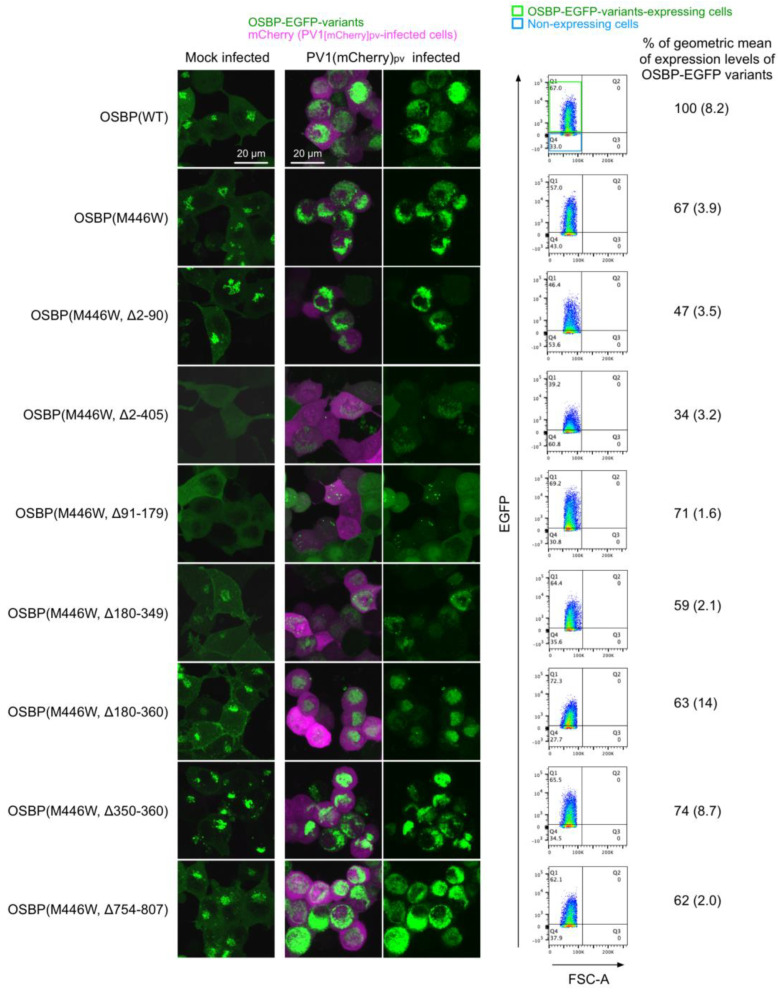
Subcellular localization and expression levels of the OSBP variants. (**Left**) Maximum projection view of HEK293 cells expressing the OSBP variants analyzed by confocal microscopy. The cells were infected with PV1(mCherry)_pv_ at an MOI of 50 at 37 °C for 5 h. Images of the representative cells (mock-infected or PV1(mCherry)_pv_-infected cells) at 5 h p.i. are shown. (**Right**) Expression levels of the OSBP variants. The cells were analyzed by flow cytometry. The expression level of OSBP-EGFP(WT)-expressing cells is taken as 100%. The data are representative of two independent experiments with two or three biological replicates.

We analyzed expression levels and subcellular localization of the OSBP variants (Figure 2). Flow cytometry analysis suggested that about 70% of the cells expressed the variants, except for the Δ2–90 or Δ2–405 variants, which were apparently expressed in about 40% of the cells, possibly because of the low expression levels. Western blot analysis confirmed expected molecular mass of each variant and showed generally good correlation with the flow cytometry analysis in terms of the expression levels (Figure S1). Under the non-infected condition (mock-infected cells), the full-length WT and the M446W variant with no deletion localized at the Golgi and cytoplasm. The variants that lack the PHD (Δ2–405 and Δ91–179 variants) localized in the cytoplasm and also in the nucleus for the Δ2–405 variant, but not at the Golgi. The variants that lack a CC region (Δ180–349 and Δ180–360 variants) localized at the plasma membrane in addition to the cytoplasm and the Golgi (Figure S2). The variant that lacks only the FFAT motif (Δ350–360 variant) showed enhanced localization at the Golgi. Deletion of C-terminal aa 754–807 had no effect on the subcellular localization compared to the full-length M446W variant. Under the infected condition (PV1[mCherry]_pv_-infected cells), the full-length WT and the M446W variant with no deletion relocalized at the replication organelle (RO) in a perinuclear region from the cytoplasm and the Golgi. Except for the variants that lack the PHD (Δ2–405 and Δ91–179 variants), other variants showed similar relocalization to that of the full-length M446W variant; the Δ2–405 variant showed faint relocalization to the RO and in the cytoplasm, and the Δ91–179 variant showed dot-like localization in a perinuclear region or the cytoplasm. These results suggested that PHD is the primary determinant of targeting of OSBP to the RO.

**Figure 3:**
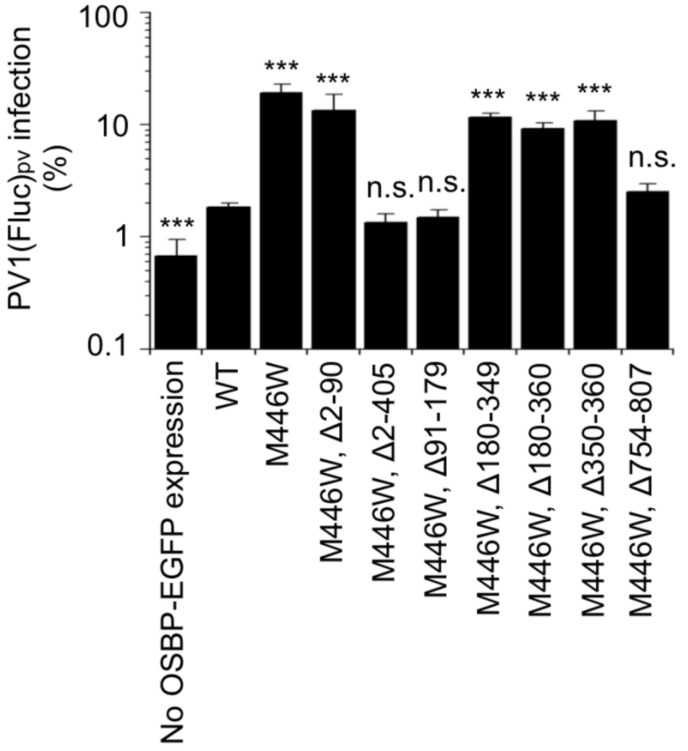
Functional dominance assay of OSBP. HEK293 cells expressing each of the OSBP variants were infected with PV1(Fluc)_pv_ at an MOI of 0.5 at 37 °C for 7 h in the presence (20 μM) or absence of T-00127-HEV2. PV1(Fluc)_pv_ infection in the absence of T-00127-HEV2 is taken as 100%. Statistical significance of the infection to that in the WT-expressing cells was shown. n.s., not significant. ***, *p* < 0.001. The data represent the mean and standard deviation of two independent experiments with two or three biological replicates.

We performed a functional dominance assay for these cell lines by using an OSBP inhibitor T-00127-HEV2 [6,26]. T-00127-HEV2 inhibits the sterol transfer activity of endogenous OSBP, while those of the M446W variants could show tolerance. In this assay, significant viral replication was observed in the cells expressing the full-length M446W variant, variants that lack the IDR (Δ2–90 variant), CCR (Δ180–349 variant), or the FFAT motif (Δ180–360 and Δ350–360 variants) in the presence of T-00127-HEV2 (Figure 3). In contrast, the WT OSBP, variants that lack the PHD (Δ2–405 and Δ91–179 variants) or a conserved C-terminal region (Δ754–807 variant), failed to support viral replication. These results suggested that the PHD and ORD are essential for the function of OSBP to support PV replication.

## 4. Discussions

Host PI4KB and OSBP form a functional axis to provide PI4P and accumulate UC in the RO in picornavirus-infected cells [5,6,7,31,32,33]. In a previous study, essential domains of PI4KB required for EV replication were determined by *trans* complementation assay with a *PI4KB*-knockout cell line [34]. In the present study, we identified the PHD and ORD, but not other domains (IRD, CC, FFAT), as the essential domains of OSBP to support PV replication by the functional dominance assay [26].

### 4.1. PHD and ORD

PHD of OSBP binds to PI4P and also other phosphoinositides (PIs), including phosphatidylinositol 3-monophosphate (PI3P), phosphatidylinositol 5-monophosphate (PI5P), phosphatidylinositol 3,5-bisphosphate (PI[3,5]P2), and phosphatidylinositol 4,5-bisphosphate (PI[4,5]P2) [11]. In infected cells, OSBP is rapidly released from the RO after inhibition of host PI4KB activity [8,35], suggesting PI4P as a primary determinant for the targeting to the RO. Targeting of the PHDs to the Golgi requires interaction with host ARF1 in addition to PIs [36,37], possibly due to the low specificity to PIs. Ishikawa-Sasaki et al. reported interactions of viral proteins of a picornavirus (Aichi virus) with OSBP/VAPA/VAPB/SAC1, which might serve as additional factors for the targeting to the RO [38]. The PHD might not be essential in in vitro sterol transfer activity of OSBP [39], but seems essential for the viral replication, possibly as a tether to the RO (Δ91–179 variant, Figure 2 and Figure 3).

ORD of OSBP binds its ligands (sterols and PI4P) and exchanges sterol and PI4P between cellular organelles [12,25]. Weak localization of ORD (Δ2–405 variant) in the RO was observed, which was compromised in the presence of the CCR and the FFAT motif (Δ91–179 variant) (Figure 2) [12]. The Δ2–405 variant lacks the dimerization domain (residues 261–288) [23], suggesting that the weak targeting to the RO seemed solely mediated by the ORD. As a negative control of the OSBP variants with the M446W substitution, we analyzed a putative functionally null OSBP variant (Δ754–807 variant) that lacks a structurally conserved C-terminal region among human OSBP family I, II, and III (OSBP/OSBP2, OSBPL1A/OSBPL2, OSBPL3/OSBPL6/OSBPL7, respectively) [26,40,41,42]. The Δ754–807 variant failed to support PV replication despite its relocalization to the RO, supporting the importance of this conserved region and that both the PHD and ORD are essential to support PV replication.

### 4.2. IRD, CCR, and FFAT Motif

The IRD controls orientation/mobility of OSBP at the membrane contact site (MCS) [22] (Figure 3). We observed that lack of IRD reduced the expression level of the OSBP variant (Δ2–90 variant) (Figure 2), suggesting that the IRD could have a significant role in the context of endogenous OSBP, but not in ectopically overexpressed variants.

The CCR (residues 180–349) includes the dimerization domain (residues 261–288) that is required for homo/heterodimerization with OSBP/OSBP2 [12,23]. Interestingly, the Δ180–349 and Δ180–360 variants, both of which lack the dimerization domain, showed partial localization at the plasma membrane in the HEK293 cells (Figure 2 and Figure S2). Localization of OSBP at the plasma membrane requires dimerization with OSBP2 in OSBP2-knock-in T cells [16], in contrast to the above observations in the HEK293 cells. Factors that could affect subcellular localization of OSBP remain to be elucidated, but the CCR seemed not essential for the targeting to the RO in the infected cells (Figure 2).

The FFAT motif (residues 350–360) is required for the interaction with VAPA/VAPB at the ER; thus, it is considered to be essential for the lipid transfer activity of OSBP at the membrane contact site between the ER and the *trans* Golgi [24]. In fact, double knock-down of VAPA/VAPB caused significant suppression of Aichi virus replication [38]. Therefore, it was a surprise that the Δ350–360 variant could support PV replication (Figure 3). The RO of picornavirus derives from the ER [43,44,45], so VAPA/VAPB should locate in cis to the viral replication complex on the same membrane. Accumulation of UC in the RO [7,8,38] and the traffic from the plasma membrane [46] were observed in the infected cells; however, the target organelles for the sterol/PI4P exchange of OSBP in the infected cells remains to be clarified.

The limitations of this study include the high expression levels of ectopically expressed OSBP variants (10- to 20-fold higher levels than that of endogenous OSBP) [26], which might aberrantly complement some functions of endogenous OSBP, and a specific antagonistic effect of T-00127-HEV2 that could inhibit sterol-transfer activity of WT OSBP, but has a lower effect on the PI4P-transfer activity [19]. Therefore, the sterol transfer activity of OSBP could be the target of the assay, but other activities of OSBP, including the PI4P transfer activity, remain to be evaluated.

## Supplementary Materials

The following supporting information can be downloaded at: https://www.mdpi.com/article/10.3390/v14122672/s1. Figure S1: Western blot analysis of the OSBP variants. Figure S2: Subcellular localization of the OSBP variants.

## Abbreviations

aa	amino acid
CCR	coiled-coil region
ER	endoplasmic reticulum
EV	enterovirus
FFAT	two phenylalanines in an acidic tract
IDR	intrinsically disordered region
ORD	OSBP-related domain
OSBP	oxysterol-binding protein
PHD	pleckstrin homology domain
PI4KB	phosphatidylinositol-4 kinase III beta
PI4P	phosphatidylinositol 4-monophosphate
PV	poliovirus
PV1_pv_	type 1 PV pseudovirus
UC	unesterified cholesterol
