# Supplementary figures and images for: Essential Domains of Oxysterol-Binding Protein Required for Poliovirus Replication
Source: Viruses. 2022 Nov 29;14(12):2672. doi: 10.3390/v14122672 (PMC9786093; doi:10.3390/v14122672)

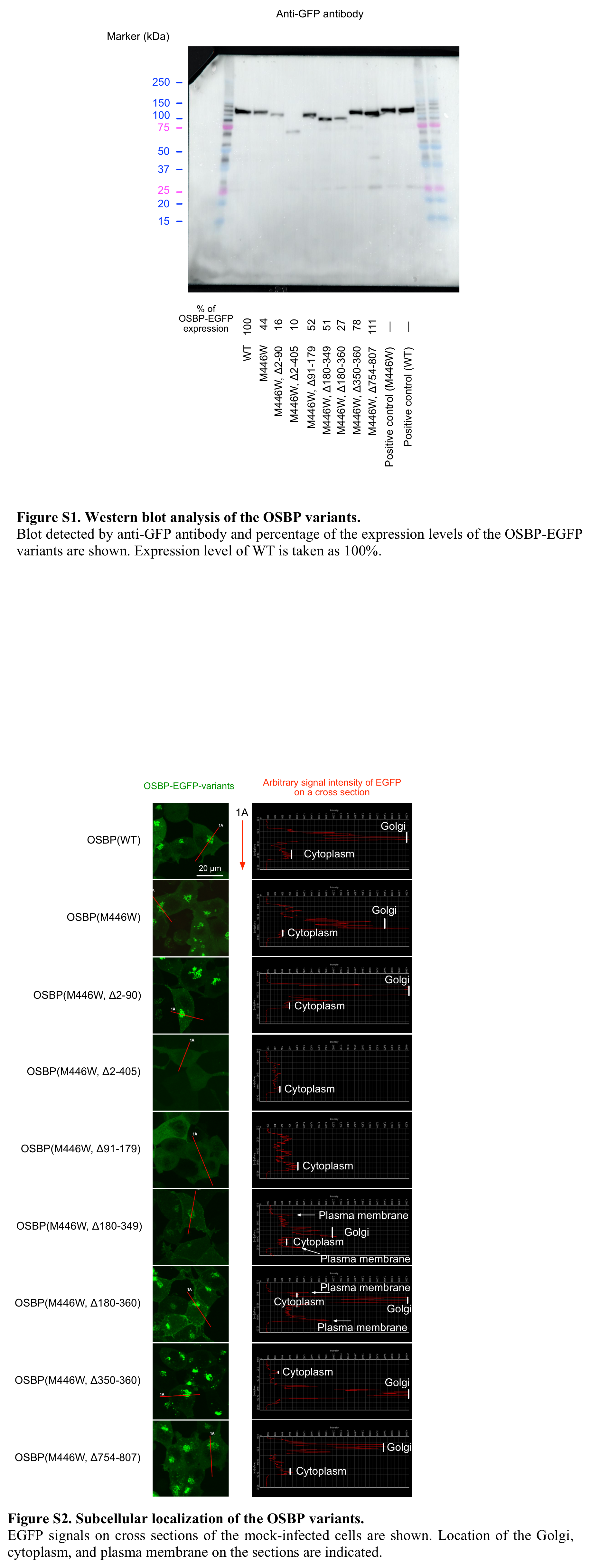

Supplement: Supplementary file 1 [file viruses-14-02672-s001.zip › viruses-2028154-supplementary.tiff]
